# Mutation of TGFβ-RII eliminates NSAID cancer chemoprevention

**DOI:** 10.18632/oncotarget.23792

**Published:** 2017-12-31

**Authors:** Juana Martín-López, Pierluigi Gasparini, Kevin Coombes, Carlo M. Croce, Gregory P. Boivin, Richard Fishel

**Affiliations:** ^1^ Department of Cancer Biology and Genetics, The Ohio State University Wexner Medical Center, Columbus, OH, USA; ^2^ Department of Biomedical Informatics, The Ohio State University Wexner Medical Center, Columbus, OH, USA; ^3^ Department of Pathology, Boonshoft School of Medicine, Wright State University, Dayton, OH, USA; ^4^ Department of Physics, The Ohio State University, Columbus, OH, USA

**Keywords:** COX-independent, cardioprotection, mismatch repair, naproxen, colon cancer

## Abstract

Non-steroidal anti-inflammatory drugs (NSAIDs) exhibit anti-neoplastic (chemoprevention) activity for sporadic cancers and the hereditary cancer predisposition Lynch syndrome (LS/HNPCC). However, the mechanism of NSAID tumor suppression has remained enigmatic. Defects in the core mismatch repair (MMR) genes *MSH2* and *MLH1* are the principal drivers of LS/HNPCC. Previous work has demonstrated that the *villin*-*Cre^+/−^Msh2^flox/flox^* (VpC-Msh2) mouse is a reliable model for LS/HNPCC intestinal tumorigenesis, which is significantly suppressed by treatment with the NSAID aspirin (ASA) similar to human chemoprevention. Here we show that including a TGFβ receptor type-II (*Tgfβ-RII*) mutation in the *VpC-Msh2* mouse (*villin*-*Cre^+/−^Msh2^flox/flox^Tgfβ*−*RII^flox/flox^*) completely eliminates NSAID tumor suppression. These results provide strong genetic evidence that TGFβ signaling and/or effectors participate in NSAID-dependent anti-neoplastic processes and provide fresh avenues for understanding NSAID chemoprevention and resistance.

## INTRODUCTION

Aspirin (ASA) has become a standard for cardio-protection and cancer chemoprevention because of its safety and effectiveness [1, 2]. In addition to its influence as a preventive [3, 4], ASA appears beneficial in reducing recurrence and chemotherapeutic resistance in patients diagnosed with breast and colorectal cancer [5, 6]. NSAID cardio-protection and chemoprevention has been historically ascribed to inhibition of prostaglandin-endoperoxide synthase 1 and 2 (COX-1 and COX-2) that convert arachidonic acid into prostaglandin and eicosanoid precursors [7]. These precursors include the cardiovascular prothrombotic activator Thromboxane A_2_ (TxA_2_) and the pro-inflammatory tumorigenesis activator Prostaglandin E2 (PGE_2_) [7].

ASA inhibits COX by irreversibly binding the enzyme [8]. Naproxen (NAP) is a propionic acid-family NSAID that also includes ibuprofen [9], both of which act as reversible inhibitors of the COX enzymes [10]. While majority of NSAIDs affect the tissue inducible COX-2, ASA, ibuprofen and NAP appear better at inhibiting the constitutively expressed COX-1 [11, 12]. Highly specific COX-2 inhibitors have been developed as 2^nd^-generation anti-inflammatory and chemoprevention drugs [13]. Paradoxically, a number of these appear to increase the risk of cardiovascular events [14].

NSAIDs have been shown to inhibit proliferation and/or induce apoptosis in multiple tumor cell lines irrespective of COX1 or COX2 expression [15]. Moreover, there is a discrepancy between the potency of COX1 or COX2 inhibition by NSAIDs and their chemoprevention efficacy [15]. These and numerous other observation have suggested that NSAID chemoprevention is COX-independent [15]. One metabolic paradigm is 15-hydroxyprostaglandin dehydrogenase (PGDH) that catalyzes the inactivating conversion of PGE_2_ to a 15-keto derivative [16]. PGDH has been identified as a tumor suppressor in human colon cancer [17] and the *Pgdh* knockout appears to modestly reduce tumor suppression by the NSAID sulindac in a mouse model [18]. Nevertheless, a definitive COX-independent chemoprevention mechanism is unknown and underlines the complexity and enigmatic nature of NSAID tumor prevention.

Mutation or epigenetic silencing of the human mismatch repair (MMR) genes *MSH2*, *MSH6*, *MLH1* or *PMS2* is the cause of Lynch syndrome or hereditary non-polyposis colorectal cancer (LS/HNPCC) as well as 10-40% of sporadic cancers [19]. MMR is highly conserved throughout biology and mainly processes polymerase misincorporation errors [20]. Mutation of *MSH2* or *MLH1* is associated with a more aggressive LS/HNPCC phenotype that displays a well-known genomic instability that drives tumorigenesis, which is diagnostically recognized by length alterations in simple repeat sequences (microsatellite instability or MSI) [21].

Inactivation of the transforming growth factor-β receptor type-II (*TGFβ-RII*) gene occurs in ~80% of LS/HNPCC tumors as a result of an altered coding sequence microsatellite (*BAT-RII*) [22]. The TGFβ pathway has recognized roles in regulating growth control during the early stages of tumorigenesis as well as the tumor microenvironment and metastasis in later stages of tumorigenesis [23, 24]. These observations suggest a synergistic relationship between the MMR and *TGFβ-RII* mutations in tumorigenesis [25], although a simple genetic analysis of this hypothesis has not been reported. Intriguingly, exogenous application of the TGFβ ligand appears to induce the expression of PGDH in an early-stage human carcinoma cell line [17].

## RESULTS

### Dietary naproxen dramatically increases the survival of LS/HNPCC mice

Previous studies suggested that ASA treatment induced a COX-independent selection for microsatellite-stable LS/HNPCC human tumor cells by enhancing apoptosis of the MSI subpopulation of cells [26, 27]. Moreover, dietary ASA significantly increase life span of the mouse *villin-Cre^+/−^Msh2^flox/flox^* (*VpC-Msh2*) LS/HNPCC intestinal cancer model [28] that displays all of the pathological hallmarks of human LS/HNPCC colorectal tumors [29]. These results appeared qualitatively similar to the human LS/HNPCC CAPP2 ASA chemoprevention trials [30] and supported the conclusion that the *VpC-Msh2* mouse was an effective model for LS/HNPCC tumorigenesis and ASA tumor suppression.

To determine the effect of the propionic acid NSAID family in *VpC-Msh2* mice, dietary NAP (331 ppm) was included *ad libitum* at weaning (21-25 days old) in cohorts that were clustered to reduce consanguinity. A parallel dietary exposure to ASA (400 ppm) provided a positive chemoprevention control [28]. To insure equivalent genetic backgrounds the breeding colony were refreshed by backcrossing to C57BL/6J mice prior to developing treatment cohorts (N ≥ 8; Jackson Laboratory). Treated and untreated *wild type* (*VpC^+/−^Msh2^+/+^*) mice were included to provide a comparison to the normal lifespans in these mice. All cohorts included equal numbers of male and female mice.

**Figure 1:**
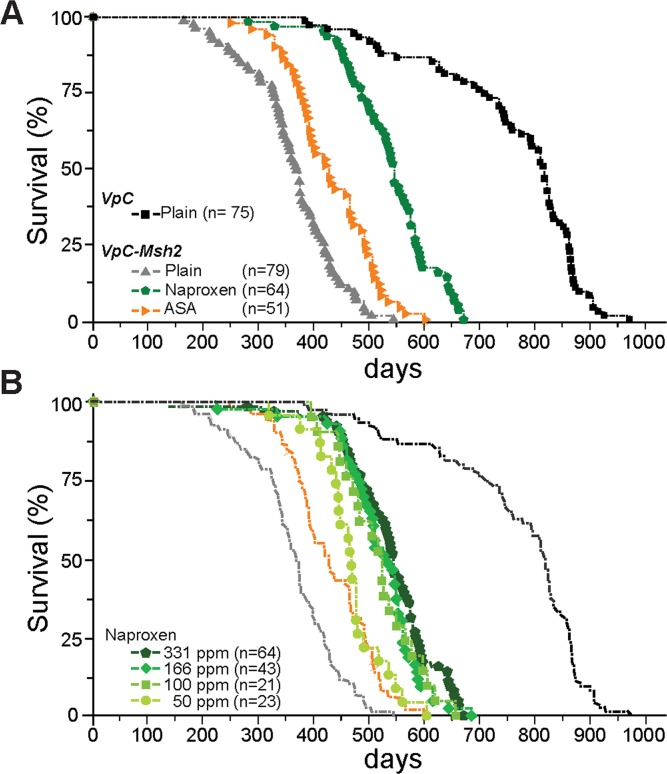
Naproxen increases the lifespan of VpC-Msh2 Lynch syndrome mice **A**. Kaplan-Meier survival curves of *VpC-Msh2* mice treated with NAP or ASA provided in food and fed *ad libitum*. *VpC-Msh2* mice treated with 331 ppm NAP (Green) or 400 ppm ASA (Orange) compared to untreated *VpC* (Black) and *VpC-Msh2* (Grey) mice. See Table 1 and Supplementary Table 1 for mean survival and statistical significance between survival cohorts. n = number of mice in cohort. **B**. Dose-dependent survival of NAP treated mice. Kaplan-Meier survival curves of *VpC-Msh2* mice treated with four different dosages of naproxen provided in food and fed *ad libitum*. From lighter to darker green color: 50, 100, 166 and 331 ppm NAP. Kaplan-Meier survival curves from (A) are shown faded in the background for comparison. See Table 1 and Supplementary Table 1 for mean survival and statistical significance between survival cohorts. n = number of mice in cohort.

The body weights of animals fed the NSAID diet was equivalent to those fed the control diet as well as the *wild type* mice suggesting that there was no overt toxicity (Table 1). As expected we found that the inclusion of dietary ASA increased the survival of *VpC-Msh2* mice by 69 days or 19% (*P* < 0.0001; Figure 1A, orange; Table 1 and Supplementary Table 1). Remarkably, a near-equivalent dose of dietary NAP increased survival of *VpC-Msh2* mice by 178 days or 49% (*P* < 0.0001; Figure 1A, green; Table 1 and Supplementary Table 1) and corresponded to 71% of the normal *wild* type lifespan (*P* < 0.0001; Figure 1A, compare green with black; Table 1) and 25% longer than ASA treated mice (*P* < 0.0001; Figure 1B, compare green with orange; Table 1 and Supplementary Table 1). While we observed no significant difference in survival in mice treated with 331 ppm, 166 ppm and 100 ppm of dietary NAP, we found 50 ppm dietary NAP reduced survival to near that of ASA treated mice (Figure 1B; Table 1 and Supplementary Table 1). These observations show that NAP chemoprevention is significantly more effective than ASA at lower doses and afforded a large experimental window for examining NSAID tumor suppression mechanisms.

**Table 1:** Survival and tumor prevalence

	Survival			Necropsy									Weight^a^ (g)	
	dose						Tumors per intestinal region^b^						100	
NSAID	(ppm)	(n)^c^	days^d^	(n)^c^	t^e^	t/n^f^	*duodenum*	*jejunum*	*ileum*	*cecum*	*colon*	*rectum*	days	Max
**Plain food**														
*Wild type*	**--**	(75)	764 ± 16	(51)	20	0.4	4 (20)	14 (70)	2 (10)	--	--	--	27 ± 6	38 ± 7
*VpC-Msh2*	**--**	(79)	362 ± 09	(62)	119	1.9	27 (22)	77 (65)	12 (10)	1 (1)	2 (2)	--	24 ± 5	32 ± 6
*VpC-Msh2-TgfβRII*	**--**	(23)	388 ± 14	(23)	67	2.9	14 (21)	39 (58)	4 (6)	5 (7)	3 (4)	2 (3)	26 ± 4	31 ± 5
**Naproxen**														
*VpC-Msh2*	**331**	(64)	540 ± 10	(50)	120	2.4	32 (27)	73 (61)	11 (9)	1 (1)	2 (2)	1 (1)	24 ± 4	35 ± 4
	**166**	(43)	521 ± 12	(33)	95	2.9	21 (22)	58 (61)	11 (12)	1 (1)	4 (4)	--	24 ± 4	33 ± 7
	**100**	(21)	519 ± 15	(17)	53	3.1	14 (26)	30 (57)	8 (15)	1 (2)	--	--	25 ± 5	35 ± 5
	**50**	(23)	468 ± 13	(22)	49	2.2	7 (14)	40 (82)	1 (2)	1 (2)	--	--	23 ± 4	34 ± 4
*VpC-Msh2-TgfβRII*	**331**	(22)	425 ± 16	(21)	40	1.9	6 (15)	19 (47)	2 (5)	2 (5)	5 (12)	6 (15)	25 ± 3	32 ± 2
**ASA**														
*VpC-Msh2*	**400**	(51)	431 ± 11	(46)	89	1.9	18 (20)	62 (70)	8 (9)	--	1 (1)	--	23 ± 4	33 ± 4
*VpC-Msh2-TgfβRII*	**400**	(23)	414 ± 19	(22)	62	2.8	10 (16)	37 (60)	7 (11)	4 (6)	--	4 (6)	25 ± 3	31 ± 4

^a^ Mean weight ± standard deviation expressed in grams. ^b^ Number in () represents the percentage of the total number of tumors; ^c^ number of mice; ^d^ Mean ± standard deviation of survival in days by Kaplan-Meier analysis, ^e^ Total number of tumors; ^f^ Average number of tumors per mouse.

### Mutation of TGFβ-RII does not significantly decrease survival of LS/HNPCC mice

**Figure 2:**
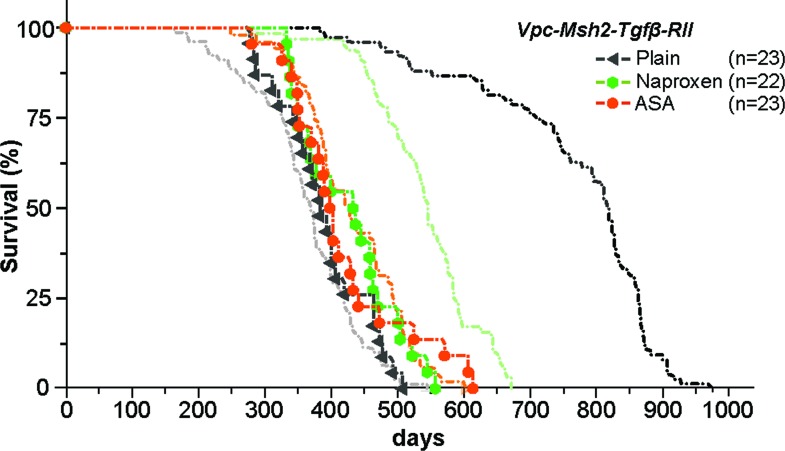
Mutation of TGFβ-RII eliminates NSAID tumor chemoprevention Kaplan-Meier survival curves of untreated (black triangle), NAP treated (331 ppm; green circle) and ASA treated (400 ppm; red circle) *VpC-Msh2-TgfβRII* mice. Kaplan-Meier survival curves from Figure 1A are shown faded in the background for comparison. See Table 1 and Supplementary Table 1 for mean survival and statistical significance between survival cohorts; n = number of mice in cohort.

**Figure 3:**
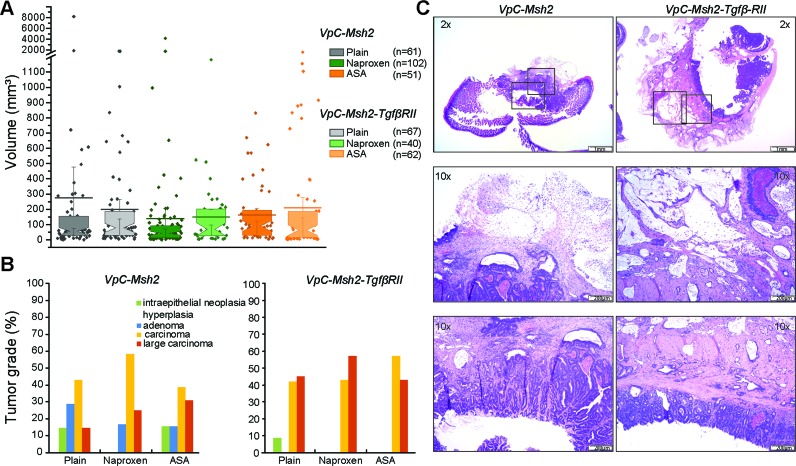
Tumors from VpC-Msh2-TgfβRII mice display increased tissue invasiveness **A**. Box plot representing tumor volume (mm^3^) of mouse cohorts in absence of treatment (grey) and treated with NAP (331ppm, green) or ASA (400ppm, orange). The number of tumors (n) is shown in parenthesis. Box plot shows median (constriction), mean (line) and upper and lower quartile (above and below constriction, respectively). **B**. Distribution of tumor grade (%) with respect to NSAID treatment for the mouse cohorts (see tumor grade color key; Supplementar*y* Table 2). Mouse genotypes are shown above graphs. **C**. Hematoxylin-eosin staining of intestinal carcinomas (scale bar; 1 mm upper panel, 200μm lower panels); Left panel, *VpC-Msh2* mutant tumor showing moderate invasiveness; Right panel, Large carcinoma with extensive invasion of the intestine muscular wall in a *VpC-Msh2-TgfβRII* tumor. Boxes indicate location of magnified (10x) areas of malignant progression.

To examine the role of TGFβ signaling in the *VpC-Msh2* LS/HNPCC mouse model we generated triple marker *villin-Cre^+/−^Msh2^flox/flox^Tgfβ-RII^flox/flox^* (*VpC-Msh2-TgfβRII*) mice, where the cre-dependent deletion of both *Msh2* and *Tgfβ−RII* was confined to the intestine from the duodenum to the rectum (Supplementary Figure 1) [29]. We found that survival of *VpC-Msh2-TgfβRII* mice was not significantly different from *VpC-Msh2* mice on a plain food diet (*P* = 0.31; Figure 2, compare black triangles with background grey line; Table 1 and Supplementary Table 1). Moreover, the tumor numbers and volumes found in the *VpC-Msh2-TgfβRII* mice were not significantly different from the *VpC-Msh2* mice observed under multiple treatment schemes (Figure 3A and Supplementary Figure 2 and Supplementary Table 1). We noted a modest shift in *VpC-Msh2-TgfβRII* tumor location to the distal bowel similar to previous reports with *Tgfβ-RII*-deficient mice (Table 1) [31]. The largely invariant tumor numbers and volumes reveal an idiosyncrasy of this mouse model in which the end-point for euthanasia under the Animal Welfare Act was almost always the result of morbid tumor-associated bowel obstruction [28, 29]. Thus, the time at which morbidity becomes critical is largely related to tumor size that is reflected in a combination of both tumor initiation and progression rates, rather than later stage cancer pathophysiology [32]. Nevertheless, we observed a significant difference in *VpC-Msh2-TgfβRII* tumor grade and serosal invasion pathology compared to tumors that arose in *VpC-Msh2* mice (*P* ≤ 0.05; Figure 3B and 3C; Supplementary Table 2 and 3). These observations are consistent with the conclusion that the *Tgfβ-RII* mutation enhances tumor aggressiveness but not overall tumor initiation and progression, or ultimately survival of tumorigenesis driven by *VpC-Msh2*.

### TGFβ-RII mutation eliminates NSAID chemoprevention

The inclusion of dietary ASA (400 ppm) or NAP (331 ppm) did not alter survival of the *VpC-Msh2-TgfβRII* mice (Figure 2, compare red and green circles with black triangles; Table 1 and Supplementary Table 1). These results provide strong genetic evidence that normal TGFβ signaling is essential for both ASA and NAP chemoprevention in this LS/HNPCC mouse model. Tumor grade and volume of ASA and NAP treated *VpC-Msh2-TgfβRII* mice appeared similar to those fed a plain diet (Figure 3A and 3B; Supplementary Table 2 and 3). We noted a shift of tumor location toward the distal bowel in NAP treated *VpC-Msh2-TgfβRII* mice similar to those mice fed a plain diet, which was less apparent with ASA treated *VpC-Msh2-TgfβRII* mice (Table 1). Importantly, tumors that arose in the NAP treated *VpC-Msh2-TgfβRII* mice displayed more serosal invasion and both ASA and NAP treated *VpC-Msh2-TgfβRII* mice presented with more desmoplasia; a tendency observed with similarly treated *VpC-Msh2* mice (Supplementary Table 1 and 2). None of the tumors, regardless of treatment, displayed significantly different tissue inflammation (Supplementary Table 2 and 3).

## DISCUSSION

The unique observation that the *Tgfβ-RII* mutation abolished both the ASA and NAP tumor suppression in the *VpC-Msh2* LS/HNPCC mouse model is a compelling indicator that TGFβ pathway components and/or effectors are required for NSAID chemoprevention. *Pgdh* is the only known TGFβ-regulated effector that might be linked to PGE_2_ metabolism and ultimately classical COX-related tumorigenesis. Unfortunately, a lack of specific reagents precluded detailed examination of *Pgdh* in TGFβ-dependent NSAID chemoprevention. Nonetheless, there are likely to be additional TGFβ pathway components involved in NSAID tumor suppression that are downstream of *Tgfβ-RII* and prior to *Pgdh* regulation that might explain the historical inconsistencies between COX-dependent and COX-independent mechanisms. One might predict that tumors in *VpC-Msh2* mice treated with ASA and NAP may have escaped NSAID chemoprevention by acquiring a mutation in *Tgfβ-RII* or some critical downstream genes in the TGFβ signaling pathways. Consistent with this idea, tumors that arose in *VpC-Msh2* mice treated with ASA and NAP appeared to exhibit a more aggressive pathology similar to tumors that arose in *VpC-Msh2-TgfβRII* mice (Figure 3B; Supplementary Table 2 and 3).

Our results appear vaguely similar to studies with the Apc^min/+^ mouse model where deletion of the GDF-15 TGFβ-superfamily member reduced tumor suppression by the NSAID sulindac [33]. However, it remains unclear how GDF-15 that is overexpressed in the liver following liver, kidney, heart or lung damage [34], might synergistically function with sulindac to reduce Apc^min/+^ tumors. Because sulindac has been linked to hepatic and reproductive toxicity [35], it is unlikely that this NSAID derivative would be useful for long-term human chemoprevention, especially with a hereditary cancer predisposition syndrome such as LS/HNPCC. Decades of safe and effective therapeutic use of NAP suggest that it might serve as a significantly better long-term cancer chemopreventive than ASA for LS/HNPCC and perhaps other sporadic cancers.

Finally, ASA resistance has been reported as a contributor to cardiovascular events in patients that regularly take low dose ASA for cardio-protection [36]. Clearly, the identification of genes associated with NSAID resistance is essential to determining ASA efficacy in patients. Recognition that reduced TGFβ signaling plays a role in resistance to NSAID chemoprevention should provide new avenues for the genetic analysis of potential contributors to ASA resistance in both cardiovascular protection and cancer chemoprevention.

## MATERIALS AND METHODS

### Mouse strains

The generation of the LS/ HNPCC transgenic mouse: *VpC^+/−^ Msh2^flox/flox^*, has been previously described in detail [28]. Briefly, Msh2*^floxflox^* mice were crossed to pVillin-Cre mice [strain B6.SJL_Tg (Vil-Cre) 997Gum/J] from the Jackson Laboratory until homozygosity was achieved. The *VpC^+/−^ Msh2^flox/flox^ Tgfβ-RII^flox/flox^* transgenic mice were obtained by crossing the strain generated above with a conditional knock-out mouse for the *Tgfβ-RII* gene (B6.129S6-*Tgfβr2^tmHlm^*) obtained from the NCI Mouse Repository (Frederick, MD). Exon 2 of the *Tgfβ-RII* gene is flanked by LoxP sites in this mouse model [37]. The *site-specific* deletion of the *Msh2* and *Tgfβ-RII* genes in the epithelia of small and large intestines results from the combination of Cre expression and LoxP sequences in similar genetic backgrounds (Supplementary Figure 1). Mice were maintained on a C57Bl/J6 background (N ≥ 8) and the deletion of both genes in the intestinal epithelial cells of the embryo had no obvious phenotype during development or following birth. Groups of *VpC^+/−^ Msh2^+/+^* mice were included as controls for potential effects of Cre expression on NSAIDs administration.

Animals were bred in a barrier one facility and maintained according to the NIH animal care and use guidelines. All animals used were genotyped by PCR for the 3 genes tested. After ten generations, mice were crossed back with WT C57Bl/J6 mice to regenerate the background. All experiments involving animals received prior approval from The Ohio State University Institutional Animal care and Use Committee (IACUC).

### Criteria for early removal, tissue collection and staining

The mouse *VpC^+/−^Msh2^flox/flox^* and *VpC^+/−^Msh2^flox/flox^Tgfβ-RII^flox/flox^* mutant strains used in this study develop sporadic intestinal tumors over their lifetime regardless of the NSAID treatment. As a consequence, the animal condition will deteriorate over time. Weekly weighing and body check was performed. If one or more of the following signs were observed, the mouse was euthanized: 1.) weigh loss (20% or more), 2.) abdominal distension, 3.) hunched posture, 4.) poor coat quality, 5.) dyspnea and 6.) enlarged swelling of the lymph nodes around the legs.

Animals were euthanized by CO_2_ inhalation, followed by cervical dislocation and pathological inspection. Tumor presence, number, location and size were annotated for each mouse when possible and intestines were stored in 10% formalin. Fixed tumors were embedded in paraffin and slides were stained with hematoxylin and eosin according to standard protocols. Tumor volume was calculated considering tumor as an ellipse: π/6 x (a+b+c) or π/6 x (a+b^2^), when height could not be measured.

### Treatment groups

Each experimental cohort was designed to contain the same ratio of males and females. Excessive inbreeding was avoided. Each group contained 20-24 mice. Animals were placed in the study at weaning and monitored over their lifetime.

### ASA and Naproxen

Mice were fed *ad libitum*. Administration of NSAIDs was established at weaning, at 22-25 days old. The doses are listed as ppm of the NSAID per powdered food. Powdered irradiated diet food (Teklad Harlan, LM-485) was mixed with 400 ppm ASA or Naproxen at 50 ppm, 100 ppm, 166 ppm or 331 ppm (Sigma-Aldrich). Mice not receiving NSAIDs were also fed with powdered food. New food was prepared on regular basis and was provided at least twice per week. Cages and bedding were changed weekly.

### Statistical analysis

All statistical analyses were performed using OriginPro 9.0, GraphPad and R software. Survival data from the Kaplan-Meier (KM) survival curves were compared with the log-rank test. In all cases a value of *P* ≤ 0.05 was considered statistically significant.

### Quantitative real-time PCR analysis

Isolation of epithelial tissue from the different parts of the intestine was performed as previously described [38, 39], and genomic DNA was extracted with DNAeasy blood and tissue kit (Qiagen). DNA concentration was assessed using a Nanodrop 2000 spectrophotometer (Thermo Scientific). For optimal results, 10ng of DNA per PCR reaction was used. QPCR was performed in a StepOne Plus Real-Time PCR System (Applied Biosystems) using iQ SYBR Green Supermix (Bio-Rad) and specific primers. Primers were designed by Primer3 software and tested with NCBI Primer Blast. Two sets of primers were designed for each gene; one set amplified in the presence of cre-mediated recombination and another one in its absence. 10ul reactions were performed in duplicate and standardized using relative amount of intron 1 product of the *Tgfβ-RII* gene as the internal control. Melting curve analysis was performed with each run to test specificity in SYBR green assays. Percentage of recombination was calculated by establishing the ratio between recombinant and non-recombinant alleles for each mouse.

## SUPPLEMENTARY MATERIALS FIGURES AND TABLES


