# Supplementary material for: Mutation of TGFβ-RII eliminates NSAID cancer chemoprevention
Source: Oncotarget. 2017 Dec 31;9(16):12554–61. doi: 10.18632/oncotarget.23792 (PMC5849154; doi:10.18632/oncotarget.23792)
Supplement: Supplementary file 1 [file oncotarget-09-12554-s001.pdf]

Mutation of TGFβ-RII eliminates NSAID cancer chemoprevention

SUPPLEMENTARY MATERIALS

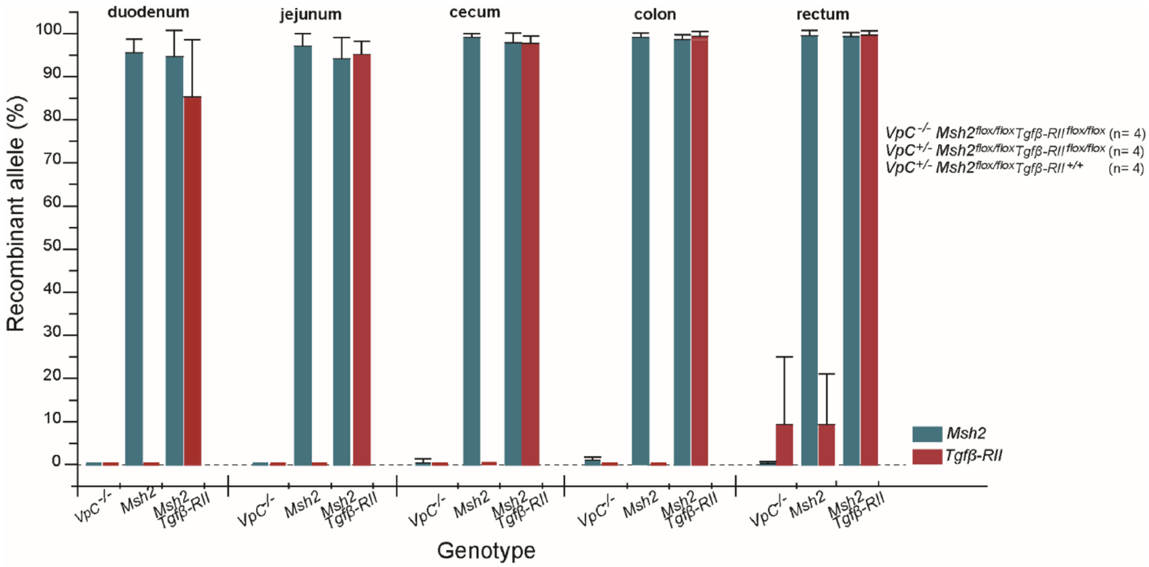

Supplementary Figure 1: Percentage of Cre-dependent gene deletion in the intestine. *VpC-Msh2-TgfβRII* (n=4), *VpC-Msh2* (n=4) and *VpC-/- Msh2-TgfβRII* (n=4) mice were analyzed by quantitative PCR for the amount of *Msh2* and *TgfβRII* recombinant alleles throughout the intestine. Percentage of *Msh2* and *TgfβRII* recombinant alleles are shown in blue and red, respectively.

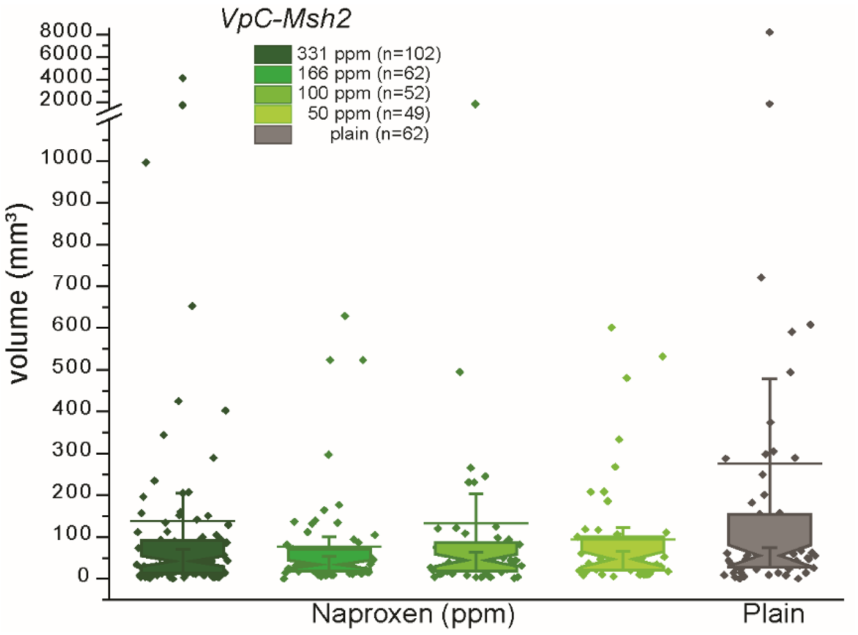

Supplementary Figure 2: Dose-dependence of Naproxen and tumor volume. *VpC-Msh2* mice were fed with four different concentrations of naproxen provided in food and fed *ad libitum*. Concentrations of naproxen from left to right: 331 ppm NAP (n=102 tumors), 166 ppm (n=62), 100 ppm (n=52) and 50 ppm NAP (n=49 tumors). Tumor volume of untreated mice are shown in grey (n=62 tumors measured).

Supplementary Table 1: Log-rank comparison among treatments and cohorts

		VpC $^{+/-}$ Msh2 $^{flox/flox}$						*VpC $^{+/-}$ Msh2 $^{flox/flox}$ Tgfβ-RII $^{flox/flox}$*		
		**Untreated**	**Naproxen**				**ASA**	**Untreated**	**Naproxen**	**ASA**
			331 ppm	166 ppm	100 ppm	50 ppm	400 ppm		331 ppm	400 ppm
VpC $^{+/-}$ Msh2 $^{flox/flox}$	**Untreated**							0.31	**0.004**	**0.02**
	**Naproxen** 331 ppm	**< 0.0001**						**< 0.0001**	**< 0.0001 *****	**< 0.0001 *****
	**Naproxen** 166 ppm	**< 0.0001**	0.19					**< 0.0001 *****	**< 0.0001 *****	**0.0002**
	**Naproxen** 100 ppm	**< 0.0001 *****	0.16	0.89				**< 0.0001 *****	**0.0002**	**0.003**
	**Naproxen** 50 ppm	**< 0.0001 *****	**< 0.0001 *****	**0.0005**	**0.01**			**0.0009**	0.09	0.17
	**ASA 400 ppm**	**< 0.0001 *****	**< 0.0001 *****	**< 0.0001 *****	**< 0.0001 *****	0.16		**0.01**	0.65	0.95
VpC $^{+/-}$ Msh2 $^{flox/flox}$ Tgfβ-RII $^{flox/flox}$	**Untreated**									
	**Naproxen 331 ppm**							0.13		
	**ASA 400 ppm**							0.27	0.91	

Supplementary Table 2: Cancer progression and tumoral grade

		VpC $^{+/-}$ *Msh2* $^{flox/flox}$			*VpC* $^{+/-}$ *Msh2* $^{flox/flox}$ *Tgfβ-RII* $^{flox/flox}$		
**Parameter**	**Grade**	***Plain***	***Naproxen***	***ASA***	***Plain***	***Naproxen***	***ASA***
**Tumor**	*Intraepithelial neoplasia/ hyperplasia*	2 (14)	0	2 (15)	1 (8)	0	0
	Adenoma	4 (29)	2 (17)	2 (15)	0	0	0
	Carcinoma	6 (43)	7 (58)	5 (39)	5 (42)	3 (43)	4 (57)
	Large carcinoma	2 (14)	3 (25)	4 (31)	6 (60)	4 (57)	3 (43)
**Serosal Invasion**	*None / Normal*	8 (57)	2 (33)	6 (46)	2 (18)	0	0
	Mild	2 (14)	1 (17)	4 (31)	2 (18)	0	3 (43)
	Moderate	0	2 (33)	1 (8)	1 (9)	0	0
	Severe	3 (21)	0	1 (8)	2 (18)	1 (17)	2 (29)
	Very Severe	1 (7)	1 (17)	1 (8)	4 (36)	5 (83)	2 (29)
**Metaplasia**	*Squamous metaplasia*	0	1 (0.08)	0	1 (0.17)	0	0
**Desmoplasia**	*None / Normal*	5 (36)	3 (25)	4 (31)	1 (8)	0	0
	Mild	1 (7)	1 (8)	5 (38)	2 (17)	0	0
	Moderate	3 (21)	4 (33)	1 (8)	1 (8)	0	1 (14)
	Severe	5 (36)	4 (33)	3 (23)	6 (50)	7 (100)	6 (86)
	Very Severe	0	0	0	2 (17)	0	0
**Inflammation**	*None/ normal*	5 (36)	3 (27)	4 (31)	1 (9)	1 (17)	0
	Mild	4 (29)	3 (27)	5 (38)	5 (45)	2 (33)	3 (43)
	Moderate	3 (21)	3 (27)	2 (15)	4 (36)	0	3 (43)
	Severe	2 (14)	2 (18)	2 (15)	1 (9)	4 (67)	1 (14)
	Very Severe	0	0	0	0	0	0

*Parenthesis indicate percentage of the total number of tumors analyzed

Supplementary Table 3: Tumor p-values obtained from t-test comparison among cohorts and treatments

Parameter			*VpC* $^{+/-}$ *Msh2* $^{flox/flox}$			*VpC* $^{+/-}$ *Msh2* $^{flox/flox}$ *Tgfβ-RII* $^{flox/flox}$		
			Plain	Naproxen	ASA	Plain	Naproxen	ASA
**Tumor Grade**	*Msh2* $^{flox/flox}$	Untreated						
		Naproxen	0.13					
		ASA	0.48	0.52				
	Msh2 $^{flox/flox}$ *Tgfβ-RII* $^{flox/flox}$	Untreated	**0.045**	0.44	0.23			
		Naproxen	**0.018**	0.26	0.11	0.53		
		ASA	**0.038**	0.52	0.20	0.80	0.63	
**Serosal Invasion**	*Msh2* $^{flox/flox}$	Untreated						
		Naproxen	0.56					
		ASA	0.90	0.47				
	Msh2 $^{flox/flox}$ *Tgfβ-RII* $^{flox/flox}$	Untreated	**0.05**	0.30	**0.032**			
		Naproxen	**0.0003**	**0.04**	**0.0001**	**0.05**		
		ASA	0.056	0.27	**0.034**	0.93	**0.037**	
**Desmoplasia**	*Msh2* $^{flox/flox}$	Untreated						
		Naproxen	0.73					
		ASA	0.49	0.29				
	Msh2 $^{flox/flox}$ *Tgfβ-RII* $^{flox/flox}$	Untreated	0.08	0.15	**0.015**			
		Naproxen	**0.012**	**0.016**	**0.0009**	**0.31**		
		ASA	**0.024**	**0.033**	**0.002**	0.47	0.34	
**Inflammation**	*Msh2* $^{flox/flox}$	Untreated						
		Naproxen	0.63					
		ASA	0.98	0.64				
	Msh2 $^{flox/flox}$ *Tgfβ-RII* $^{flox/flox}$	Untreated	0.44	0.83	0.45			
		Naproxen	0.13	0.28	0.13	0.29		
		ASA	0.23	0.48	0.24	0.51	0.62	
